# miRNAs: The Road from Bench to Bedside

**DOI:** 10.3390/genes14020314

**Published:** 2023-01-25

**Authors:** Giuseppe Iacomino

**Affiliations:** Institute of Food Sciences, National Research Council, Via Roma, 64, 83100 Avellino, Italy; giuseppe.iacomino@isa.cnr.it; Tel.: +39-0825299431

**Keywords:** miRNA, circulating miRNA, theranostic biomarkers, RNA-based therapeutic, personalized medicine, translational medicine

## Abstract

miRNAs are small noncoding RNAs that control gene expression at the posttranscriptional level. It has been recognised that miRNA dysregulation reflects the state and function of cells and tissues, contributing to their dysfunction. The identification of hundreds of extracellular miRNAs in biological fluids has underscored their potential in the field of biomarker research. In addition, the therapeutic potential of miRNAs is receiving increasing attention in numerous conditions. On the other hand, many operative problems including stability, delivery systems, and bioavailability, still need to be solved. In this dynamic field, biopharmaceutical companies are increasingly engaged, and ongoing clinical trials point to anti-miR and miR-mimic molecules as an innovative class of molecules for upcoming therapeutic applications. This article aims to provide a comprehensive overview of current knowledge on several pending issues and new opportunities offered by miRNAs in the treatment of diseases and as early diagnostic tools in next-generation medicine.

## 1. General Concepts

Recent improvements in omics research have accelerated advances in personalized medicine with a driving impact on epigenetic research. The term epigenetics refers to chemical changes in chromatin, inherited during cell division, that do not affect DNA sequence and that include hundreds of posttranslational changes in chromatin chemical composition, such as acetylation, phosphorylation, methylation, and ubiquitination [1,2].

Gene expression is an outstandingly controlled process, and the discovery of small noncoding RNAs (sncRNAs) has provided outstanding contributors to its well-specialized supervisory systems. Most synthesized RNAs are ribosomal ribonucleic acid (rRNA), transfer ribonucleic acid (tRNA), and noncoding RNAs, such as long noncoding RNAs (lncRNAs), circular RNAs (circRNAs), and sncRNAs [3]. sncRNAs are classified as small interfering RNAs, PIWI-interacting RNAs, endogenous small interfering RNAs, promoter-associated RNAs, small nucleolar RNAs, and microRNAs (miRNAs), based on their structure, assembly, and operational modes [4]. The latter have prospects as new therapeutics, with the RNA itself acting as a target or drug.

miRNAs are sncRNAs with a length of 20–24 nucleotides that control gene expression posttranscriptionally [5]. A distinct miRNA species acts by regulating one or multiple transcripts at the same time (up to hundreds), and a single mRNA species often exhibits multiple interaction sites for various miRNAs, thus generating complex regulatory circuits. As a result, even while single miRNAs commonly have a restrained effect on a particular target, their action produces cumulative effects by negatively influencing different transcripts in a signalling network. Accordingly, metazoan miRNAs have been dubbed the sculptors of the cell transcriptome [6].

These short molecules work as gene silencers by way of base-pairing to a target mRNA in the 3′ untranslated sequence even though they have been observed to bind anywhere alongside the mRNA sequence. Interestingly, miRNAs can act either by influencing the stability and degradation of the target mRNA or by suppressing its translation. Because they are essential for base pairing with a target mRNA, the nucleotides at positions 2–8 of a mature miRNA have been termed a "seed sequence"; however, a fraction of miRNA-target recognition is also mediated through noncanonical seed-like consensus sequences. In addition, seed-region identity has also been used to classify miRNAs into families that have a common capacity to recognize sets of target mRNAs [7]. 

Currently, 3012 unique human miRNAs have been recognized and stored in the miRTarBase repository, with a huge, estimated capacity of 4,475,477 potential miRNA–target interactions [8]. Furthermore, several issues have been reported regarding the quality of miRNA annotations in publicly available databases, as well as the reproducibility of microRNA studies [9]. Comprehensively, endogenous miRNAs have been reported to regulate most, if not all, protein-coding genes in mammals. However, with hundreds of conceivably miRNA–mRNA interactions, the human miRNA targetome is far from being determined [10]. 

The miRNA biogenesis pathway is accountable for the handling of a pre-miRNA to a mature miRNA and has been widely reported [11,12,13]. Of note, miRNAs are themselves sensitive to transcriptional and posttranscriptional control, and emerging approaches for the direct mapping of RNA modifications in sncRNAs have been proposed, with an emphasis on mass spectrometry and nanopore technologies [14]. As an example, the terminal nucleotidyltransferases (rNTrs) can modulate miRNA sequences by adding nontemplated nucleotides to the 3’-end (tailing). Accordingly, miRNA isoforms with diverse 3′-ends (and possibly functions), known as 3′ isomiRs, have been thoroughly detected by NGS [15]. The editing by adenosine deaminase acting on RNA (ADAR) is a relevant regulatory mechanism acting at the posttranscriptional level and is increasingly found in metazoans [16]. The enzyme induces A-to-I RNA editing modification of double-stranded RNA. Editing leads to changes in miRNA processing, as well as the editing of a target mRNA, thus upsetting the mutual complementarity and driving a microRNA–mRNA interactional redirection, distressing diverse cellular processes. Of note, the editing activity of ADAR is increasingly reported to be enhanced in cancer, but its influence on deriving RNA modifications is still unknown [16].

Excitingly, several endogenous cellular RNAs, such as circular RNAs, long noncoding RNAs, transcribed pseudogenes, and mRNAs, act as natural miRNA sponges, able to firmly interact with and disrupt target miRNAs, thus distressing their controlled networks [17].

Although a small number of miRNAs have a tissue-specific distribution, most reveal a broad tissue localization. Given the pervasive presence of nucleases, it is assumed that RNA molecules are prone to rapid degradation in the extracellular environment. Nevertheless, miRNA molecules have been found to retain unexpected stability in plasma and other physiological fluids since they can be packaged from cells in the form of microvesicles and exosomes or protein complexes, such as Argonaute 2 (AGO-2) (taking part in the RNA silencing complex), nucleophosmin-1 (NPM-1) (an RNA-binding protein involved in ribosome nuclear export), or high-density lipoproteins. These forms are able to be actively released into the extracellular space and reach recipient cells, where they can influence the translation of target genes, thus defining an explicit role of miRNAs in cell–cell communication [18]. Accordingly, the discovery of about 300 circulating miRNAs (c-miRNAs) has highlighted miRNAs’ potential as intercellular signalling molecules and disease biomarkers [19] since a dysregulated expression of miRNA can unsettle tightly controlled RNA networks in tissues and organs, potentially contributing to abnormalities [20]. Nevertheless, most of the functional significance of c-miRNAs remains unknown to date, and the gap between discovery and function has yet to be bridged.

Many studies have found correlations between altered levels of miRNA and the physiopathology of numerous processes including mitochondrial [21], cardiovascular [22], neurodegenerative [23], immune disease [24], inflammatory [25], rare genetic disorders, and more; most of these studies have focused on miRNAs as biomarkers of cancer [26,27,28,29,30], emphasizing their relevance as personalized theranostic factors. Interestingly, miRNAs are not only prospective markers of the onset and progression of neoplastic disease, but also indicators of responses to cancer therapy, as well as their relationship to drug resistance and the modulation of responses to cancer treatment [31].

By controlling crucial metabolic processes, miRNAs have also been found to play a role in energy balance and the oversight of metabolic pathways in living organisms [20,32,33,34,35,36,37,38]. Furthermore, the treatment of infectious diseases has been linked to several miRNAs [39]. Yet, it has been discovered that miRNA expression changes in response to dietary and lifestyle factors [40]; consequently, the recent nutrimiRomics research focuses on the impact of diet on miRNA levels in the human body, as well as their downstream impact on gene expression and subsequent health outcomes. Finally, there is growing evidence that dietary miRNAs may survive digestion [41]. However, the roles and capabilities of food-related miRNAs in modulating interspecies RNA are still an enigma.

## 2. Technological Advances Offer New Opportunities for miRNAs in Translational Medicine

Since the first report of a noncoding RNA identified in *C. elegans* in 1993 [42], the bulk of the 153,210 papers on miRNA research listed in PubMed (January 2023) reveals their critical impact on human diseases. The substantial translational interest is confirmed by searching Google’s patent database; a search for the keywords “microRNA” and “biomarker” yielded 56,021 results (as of January 2023). The quantity and diversity of studies are evocative of the astonishing complexity and limitations of miRNA research that is increasingly projected toward next-generation medicine. According to the unusual levels of miRNAs found in some unhealthy conditions, novel molecular diagnostic strategies are expected, deeply encouraging researchers and producers to focus on miRNAs as relevant noninvasive disease biomarkers. However, so far, there is no agreement on specific miRNAs suitable for early disease detection, even in crucial topic areas such as in vivo cancer research [43]. Current limitations in this discovery field are related to c-miRNA recognition methods, which must be extremely specific and able to recognize small quantities of target molecules, while also taking into account the presence of unwanted contaminants and inhibitors that could interfere with downstream analytical methodologies. Differences in all the critical steps used for miRNA extraction, detection, data normalization, validation, target gene identification, and experimental design are responsible for at least some of the discrepancies between the different studies [44]. Overall, these constraints require the timely development of standard procedures and well-recognized guidelines to accurately decipher the power and versatility of miRNAs in molecular diagnostics [45,46].

Furthermore, the prompt increase of RNA drugs in recent research and clinical development, driven in part by the success of messenger RNA vaccines in the fight against the SARS-CoV-2 pandemic, has spurred the pursuit of mRNA-based drugs for treating other conditions.

Therapeutic agents are classically based on synthetic small molecules, monoclonal antibodies, or large proteins. Traditional drugs may fail to hit intended therapeutic targets due to the inaccessibility of active sites in the target’s three-dimensional structure. RNA-based therapies can offer an exceptional opportunity to potentially reach any relevant target with therapeutic purposes. Additionally, techniques based on nucleic acid technologies can avoid the need for laborious synthesis procedures. Yet, the recognition sequence can be quickly revised and adapted to the target. Nevertheless, RNA-based therapies have specificity problems that carry the risk of side effects [47,48]; in addition, RNA-based drugs’ susceptibility to degradation may determine poor pharmacodynamics [47,48]. A number of these issues can be mitigated by chemical changes in the structure of the synthetic polymer (RNA or DNA). Of note, RNA-based drugs are usually larger in size than small-molecule therapies and carry electrical charges, which makes their intracellular delivery in their native form difficult.

In this context, the therapeutic potential of miRNA treatments is receiving attention in clinical trials of almost all human diseases [43,49]. Given the ability of miRNAs to target specific mRNAs, inhibitors based on the sequence of overexpressed miRNAs can be used prospectively in the form of anti-miRs to lower elevated levels of miRNAs and, in turn, restore their downregulated transcripts [50]. This opportunity for medically-based interventions is closely linked to the pioneering use of antisense molecules, the first molecular drugs inspired by the target sequence [51]. These synthetic tools have a sequence complementary to a specific mRNA whose levels they can modulate [52]. Anti-miRs are typically based on first-generation antisense single-stranded oligonucleotides (ASOs), or their chemically modified forms as blocked nucleic acids (LNAs), which are opportunely designed to recognize target mRNAs. Yet, anti-miRs with a 2ʹ-O-methoxyethyl substitution are classified as antagomiRs [53]. Furthermore, miRNA mimics are chemically synthesized, double-stranded small RNA molecules which mimic mature endogenous miRNAs (miRNA replacement therapy) after transfection into cells, the action of which is aimed at replacing downregulated or missing miRNA expression [54]. 

**Figure 1:**
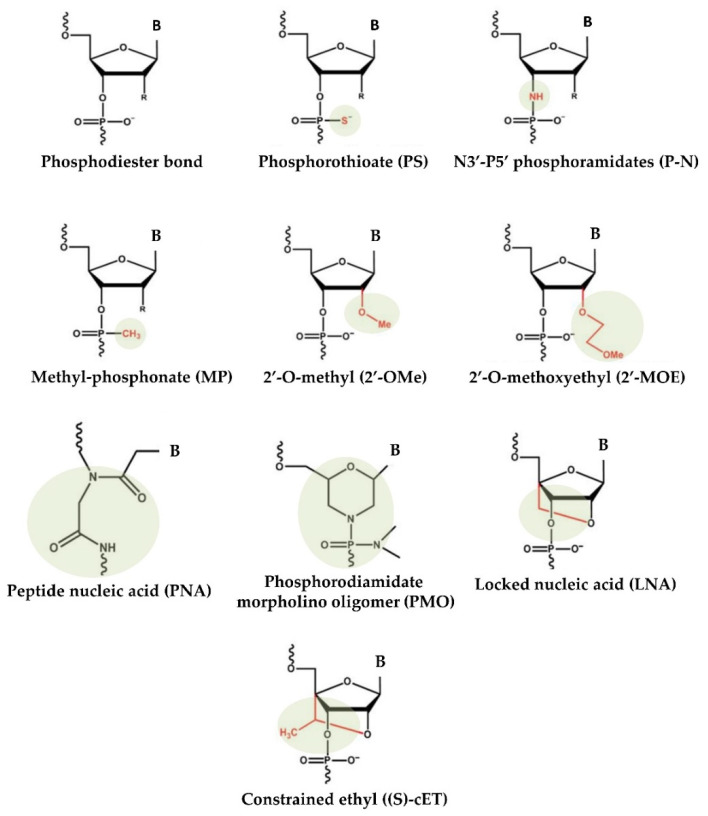
Chemical changes of oligonucleotides used in main clinical trials.

The delivery of virus-mediated miRNA-based therapies has shown great success in animal models, where adenoviruses have been successful in delivering both anti-miR and miRNA mimics. However, despite the high efficiency of virus-based miRNA delivery systems, several concerns continue, including toxicity, immunogenicity, and insert-size constraints [55]. To overcome these difficulties, over the years, nonviral approaches for the production and delivery of miRNA-based drugs have emerged. Substantial improvements in designing, synthesis, binding affinity, stability, and target modulation effects of both miRNA mimics and anti-miRs have been accomplished through chemical changes to the nucleotide backbone since a major challenge for RNA-based therapeutic strategies is the possible degradation of oligonucleotides by RNases in extracellular and endocellular compartments. Accordingly, the oligonucleotide chemistry has been widely altered by modifying the nucleotides, introducing base modifications, modifications on ribose moiety, and modifications to the phosphate group in the sugar−phosphate backbone (Figure 1). As an example, by introducing methylation (2ʹ-O-methoxyethyl), locked nucleic acids (LNAs), a nucleic acid analogue that contains a 2′-O-methoxyethyl, 2′-fluoro and 2′,4′-methylene bridge, peptide nucleic acid (PNA) a synthetic polymer, an analogue to DNA and RNA, in which the sugar–phosphate backbone has been substituted by a unit of N-(2-aminoethyl) glycine, adding phosphorothioate (PS)-like groups (by replacing the nonbridging oxygen in the phosphate group with sulphur) [51], and others [56]. Overall, these changes have been shown to increase the stability of oligonucleotides by making internucleotide linkages more resistant to the degradation of nucleases, and preserving both the ability to activate RNase H (the enzyme responsible for mRNA target cleavage) and their function in suppressing the target gene. In this research field, the diverse anti-miRNA oligonucleotide chemistries were also directly compared, and the in vitro results showed that the combination of 2′-O-methyl and LNA with phosphorothioate ends was about 10 times more active than modification alone, and 2 times as effective as the 2′-O-methyl with LNA changes [57]. Overall, up to now, most efforts to develop therapies based on chemically modified miRNAs have been directed toward the development of anti-miRs using LNA chemistry, whereas currently commercially available miRNA mimics are often modified by methylation [58,59].

Another strategic approach facilitating RNA-based therapeutics in clinical application consists of encapsulating miRNA mimics or anti-miRs in carrying vehicles to confer to them protection from nuclease.

Therapeutic miRNAs include negatively charged polymers that cannot directly cross cell membranes. To get to their intended targets, they need appropriate formulations, including carriers as well as chemical modifications. Accordingly, delivery systems must shield the therapeutic RNAs from serum nucleases, avoid immune-system interference, escape unintended interactions with serum proteins, and prevent renal clearance when given systemically. Previous studies have shown that therapeutic RNAs administered locally or topically can circumvent complications associated with systemic administration, have higher bioavailability, and have a reduced clearance as compared to those administered systemically; nevertheless, this delivery opportunity is restricted to tumours, mucous membranes, eyes, and skin [60]. In this context, the subcutaneous administration of naked mRNA, as compared with mRNA-loaded nanoparticles, leads to a more effective translation of the protein [61].

The nanocarrier encapsulation strategy can both protect and deliver the drug to recipient cells, while biological obstacles, such as immunogenicity and nuclease, are often approached by chemically modifying the nucleotide structure [62]. Various transport methods are increasingly being used to improve bioavailability, including liposomes and biodegradable polymers. In this context, liposomes, long used as immunological adjuvants in vaccination, adequately fulfil this role [63]. 

**Figure 2:**
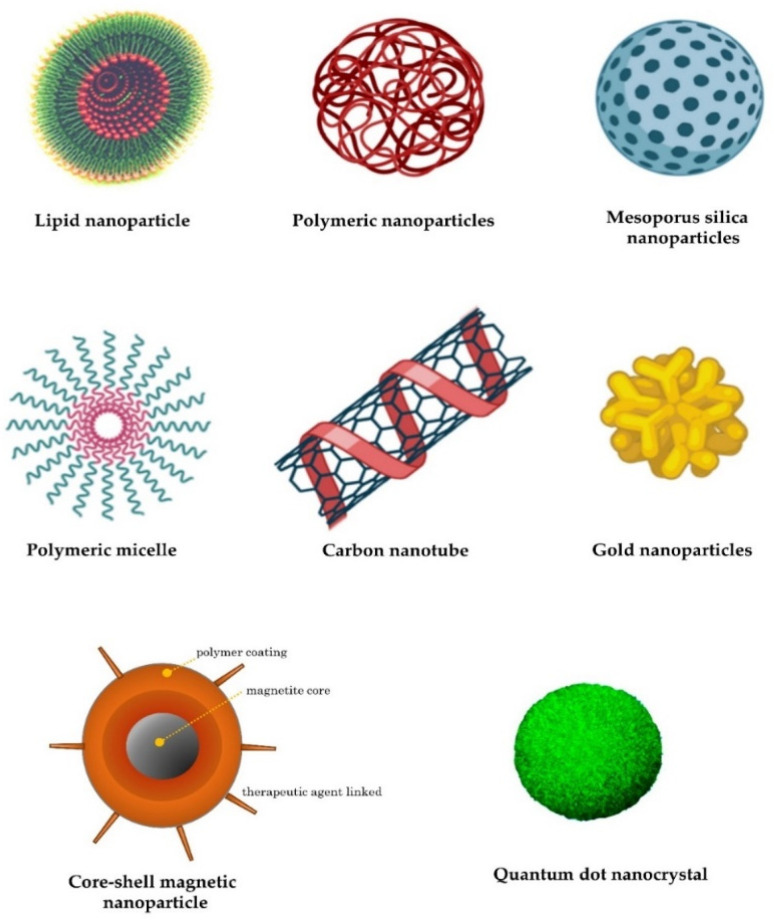
Examples of nanocarriers as drug-delivery systems.

Lipid nanoparticles are the most commonly used nonviral delivery technology for nucleic-acid-based drugs and vaccines [64]. They are composed of complexes of anionic nucleic acids and synthetic cationic lipids. The cationic lipids characteristically include amine derivatives, such as primary, secondary, and tertiary amines, in addition to quaternary ammonium, amidinium salts, and various amine combinations [65]. Furthermore, guanidine, imidazole groups (pyridinium, piperazine), and amino acids, including tryptophan, lysine, arginine, and ornithine, have also been employed [66]. A comprehensive list of commonly used cationic lipids in drug formulations was recently reviewed [66]. The advantages of lipid-based transfer systems consist of ease of assembly, biodegradability, the ability to shield nucleic acids entrapped by nucleases, the protection of renal clearance, the ability to promote cellular uptake, and the avoidance of endosomes. Additional carriers with biodegradability, biocompatibility, and low toxicity are employed. Polymeric nanoparticles are among the most-studied carriers and include synthetic and natural cationic polymers, as well as polyethylenimine (PEI), cyclodextrin, chitosan, and poly (lactic-co-glycolic acid) (PLGA) [67]. Furthermore, dendrimers, carbon nanotubes, peptides, gold nanoparticles, silica-derived nanoparticles, iron-oxide-based magnetic nanoparticles, and other nanoparticles are increasingly being studied for medical interventions (Figure 2) [62].

## 3. RNA Therapeutics

The great expectation in the field of miRNA therapeutics is fully evidenced by the number of studies discovered by querying the keyword "microRNA" in the *Clinical Trials Database* (U.S. National Library of Medicine; http://www.ClinicalTrials.gov, accessed on 20 January 2023, a search of which yielded 1213 results, a result that includes 368 studies in the recruitment or not-yet-recruited phase, 91 ongoing studies, and 414 completed studies. In this dynamic field, biopharmaceutical companies are increasingly engaged, and ongoing clinical trials point to anti-miR and miR-mimic molecules as an innovative class of drugs for upcoming therapeutic applications in the coming years [68]. As an example, in 2020, the North American market accounted for more than 45% of the comprehensive miRNA market, given the presence of key players with research and development abilities and recognized research infrastructure in terms of genomics, proteomics, oncology, and in silico tools. Interestingly, this market, estimated at USD 225.5 million in the year 2020, is projected to increase at a compound annual growth rate of 13.8% from 2020 to 2027, to reach a revised value of USD 556.1 million [69]. 

### The Lesson of Antisense Technology

The road to development and regulatory approval of emerging miRNA-based therapeutics could be faster than conventional drugs. However, such therapy, as with miRNAs in the biomarker field, is still in the early stages of development, and many aspects, including synthesis, purification, chemical stability, immunogenicity, bioavailability, distribution, metabolism, and body-elimination capacity, need to be further confirmed in preclinical studies [70]. Yet, attention must be paid to the accurate delivery of these novel drugs to target tissues and organs. Furthermore, miRNA-based drug formulations are largely based on the knowledge of antisense drugs previously approved by the U.S. Food and Drug Administration (US FDA) for the treatment of several diseases, some relevant examples of which are given below.

*Eteplirsen (Exondys 51®)*

It is a drug used to treat some types of Duchenne muscular dystrophy (DMD), caused by specific mutations in the dystrophin gene. The gene deletion determines a reading frameshift causing an early stop codon that prevents translation to functional dystrophin protein by nonsense-mediated RNA decay [71]. Exon skipping may be induced by Eteplirsen, a phosphorodiamidate morpholino oligomer (PMO), that, by selectively binding to exon 51 of the dystrophin pre-mRNA transcript, restores the reading frame phase and allows the assembly of a modified functional protein. The drug only targets specific mutations and can be used to treat about 14% of DMD cases [71].

*Fomivirsen (Vitravene)*

It was the first antisense drug to be approved by the FDA (in 1998) and by the European Agency for the Evaluation of Medicinal Products (in 1999), and it is used to treat the symptoms of retinitis, the most frequent manifestation of cytomegalovirus (CMV) disease in patients with HIV infection [72]. Fomivirsen is a phosphorothioate oligonucleotide (PS) with potent antiviral properties developed by Ionis Pharmaceuticals and marketed by Novartis CIBA Vision. When injected into a human eye, hybridization of this antisense ODN to CMV mRNA avoids RNA transcription from the immediate-early region-2 gene, thereby limiting viral replication [72]. Furthermore, this agent prevents the adsorption of CMV into host cells via a sequence-independent mechanism.

*Givosiran (Givlaari®)*

It is a drug used to treat acute hepatic porphyria (AHP) in both adults and adolescents. AHP is an uncommon genetic disorder caused by inborn defects in metabolism, in which the enzyme delta-aminolevulinate synthase 1 (ALAS1), involved in the synthesis of the heme group, is produced in excess. This condition results in a harmful accumulation of porphyrins in the liver and bone marrow that triggers a wide range of highly debilitating symptoms [73]. Givosiran, developed by Alnylam Pharmaceuticals, is a siRNA drug conjugated to GalNAc to accomplish liver-specific distribution, where it leads to a decrease in the ALAS1 enzyme and downstream metabolites [73].

*Golodirsen (Vyondys 53^TM^)*

It is an antisense PMO oligomer from Sarepta. The drug is used in DMD patients with a definite mutation in the dystrophin gene that can be treated by skipping exon 53. The objective of exon skipping is to allow the muscle tissue to make a shorter form of the modular dystrophin protein. In subjects treated with Golodirsen, levels of dystrophin increased by more than 15 fold [74].

*Inotersen (Tegsedi®)*

It was originally developed by Ionis Pharmaceuticals in 2018 and marketed globally by Akcea Therapeutics. Inotersen consists of an ASO with PS modifications along the sequence and five 2’-O-MOE nucleotides at the 5’ and 3’ ends, designed to inhibit TTR production [75]. hATTR amyloidosis is an inherited, progressive, and fatal disease for which there are limited treatment options. The mutated protein forms amyloid deposits, which accumulate in tissues and organs throughout the body, including nerves, interfering with their functions. Inotersen reduces transthyretin production, thereby decreasing amyloid formation and alleviating the symptoms of hATTR amyloidosis. Inotersen is effective in treating stage 1 or stage 2 neuropathy in patients with this condition.

*Milasen*

It is a personalized drug that was explicitly designed for the treatment of a 6-year-old child suffering from neuronal ceroid lipofuscinosis 7 (CLN7) [76]. Neuronal ceroid-lipofuscinosis (NCL) includes a group of genetic, lysosomal storage disorders characterised by progressive intellectual and motor deterioration, vision loss, seizures, and early death [77]. The genomic analysis of the patient recognized the insertion of SVA (SINE-VNTR-Alu) retrotransposon into the MFSD8 gene. The 22-mer ASO contains 2’-O-MOE- and PS-based modifications and targets cryptic splice sites in the MFSD8 pre-mRNA, restoring normal splicing (exons 6–7). Of note, Milasen treatments significantly improved the patient’s quality of life.

*Nusinersen (Spinraza®)*

Spinal muscular atrophy (SMA) represents one of the most common genetic diseases responsible for infantile death due to mutations in the SMN1 gene and the consequent injury of motor neurons. The intronic splicing silencer N1 represents a relevant pharmacological target, and several ASOs are being developed to include exon 7 in the mature mRNA transcript of the SMN2 gene to increase the synthesis of spinal motor neuron proteins. Nusinersen was developed by Biogen and contains both 2’-O-MOE- and PS-based changes [78]. The drug was approved by the FDA in 2016 and shown to be effective in improving motor function and survival in infant- and childhood-onset SMA.

*Patisiran (Onpattro®)*

It is sold under the brand name Onpattro and was developed by Alnylam. It is the first siRNA-based drug used for the treatment of polyneuropathy in people with hereditary transthyretin (TTR)-mediated amyloidosis, a rare and fatal condition [79]. TTR is a protein involved in the carriage of thyroxin and retinol-binding protein vitamin A complex. Mutations in the TTR gene produce proteins more prone to misfolding and collapse as TTR amyloid fibrils in the extracellular spaces of several organs, finally leading to their dysfunction. In a phase III study, patients treated with Patisiran displayed an 80% reduction of TTR levels in serum.

## 4. miRNA-Based Therapeutics

The development and commercialization of new diagnostics and therapeutics is a long process. Since the association of miRNAs with human disorders was discovered in 2002, their great potential as next-generation drugs has prompted fields at the interface of biology, chemistry, and medical science to invest heavily in the development and exploitation of miRNA-based therapies. However, despite the considerable number of preclinical studies, the development of both miRNA diagnostic and therapeutic applications is still in its infancy, and only a minor number of miRNA-based therapies have moved on to clinical development. In this scenario, several biotech and pharmaceutical companies have included miRNAs in their line of development to rapidly bring noncoding RNAs from the bench to the patient’s bedside, working on two types of drugs: antagomiRs and miRNA mimics. The list of miRNA-based drugs in clinical trials is expanding daily, with several candidates directed against genetic, metabolic, and oncological diseases reaching the clinical-trials stage [56,80].

*Miravirsen*

It is the first antisense miRNA that passed in clinical trials as a targeted therapy for the treatment of hepatitis C virus (HCV) infection. The drug consists of a 15-mer LNA-PS-modified ASO able to target miR-122, which controls HCV replication in the liver [81]. The long-term efficacy and safety of Miravirsen in patients with chronic HCV infection were evaluated in a phase II study. The drug was supplied to the patient through subcutaneous injection during the study. Treatment with Miravirsen has been established to determine a dose-dependent reduction in viral cargo in chronic HCV subjects. However, severe side effects halted the trial.

*RG-012*

Regulus Therapeutics has developed an anti-miR-21 for the management of Alport syndrome, an inherited disease caused by mutations in the COL4A3, COL4A4, and COL4A5 genes that result in eye abnormalities, hearing loss, and kidney disease. In this context, glomeruli become less able to function as the disease progresses, resulting in end-stage renal failure. The disease has no approved therapy [82]. miR-21 is a noncoding RNA that negatively controls genes/networks and has been described as being up-regulated in fibrotic kidney disease. Preclinical studies have shown that treatment with an anti-miR-21 significantly attenuates kidney failure by reducing the rate of progression of renal fibrosis. RG-012 has been granted orphan drug status in the United States and Europe.

*Cobomarsen (MRG-106)*

miR-155 is up-regulated in several lymphoma subtypes, as well as in diffuse large B-cell lymphoma [83]. Cobomarsen is an LNA-based antagomiR targeting miR-155. The molecule developed by miRagen Therapeutics (Viridian Therapeutics Inc) is currently in phase II trials for the treatment of cutaneous T-cell lymphoma, T-cell lymphoma, and leukaemia. 

*MRG-107*

MRG-107 is also being developed by miRagen Therapeutics and, similar to MRG-106, it targets miR-155. The miRNA plays relevant functions in the immune mechanisms and inflammation processes in amyotrophic lateral sclerosis (ALS). In the spinal cords of ALS patients, levels of miR-155 are increased. The inhibition of miR-155 has alleviated symptoms and extended survival in preclinical models of the disease.

*MRX34*

miR-34a is a natural tumour-suppressor expressed at reduced levels in many tumour types. Clinical studies have reported a negative correlation between reduced miR-34 expression and survival in several tumour types. MRX34 is a liposomal formulation of miR-34a. It is considered to be a first-in-class miRNA mimic for the treatment of a wide range of cancers, including ovarian cancer, colon cancer, cervical cancer, hepatocellular carcinoma, non-small cell lung cancer, and others. The formulation is currently in a phase I clinical trial [84].

*RG-101*

miR-122 is a liver-specific micro-RNA with relevant functions in this organ’s metabolism [20]. This miR is also an essential host factor for HCV. Clinical management with RG-101, an anti-miR-122 ODN conjugated with N-acetylgalactosamine (GalNAc) developed by Regulus Therapeutics, resulted in a significant reduction in viral loads in chronic HCV subjects. The drug is currently in a phase I clinical trial [85].

*RGLS4326*

Polycystic kidney disease (ADPKD) is an autosomal dominant disease caused by mutations in the PKD1 or PKD2 gene. (Autosomal recessive polycystic kidney disease is less common.) ADPKD represents one of the most frequent monogenetic disorders, and it is the primary genetic cause of end-stage renal disease [86]. Therapeutic opportunities for ADPKD treatment are limited. RGLS4326 is a single-stranded, chemically modified, 9-mer ASO with full complementarity to the seed sequence of miR-17. ASO potentially inhibits the pathologic functions of the miR-17 family in ADPKD [87]. This molecule is in a phase I clinical trial.

*MRG-110*

It is an antagomiR developed by MiRagen Therapeutics in collaboration with Servier. The drug targets miR-92 to treat ischemic conditions such as heart failure [88]. The phase I clinical trial is currently in the recruitment phase.

*Remlarsen (MRG-201)*

The drug is intended to mimic the activity of miR-29, a miRNA which downregulates the levels of collagen and other proteins involved in scar formation. Levels of miR-29 family members are typically downregulated in fibrotic diseases [89]. Remlarsen, an LNA RNA mimic, is being investigated to determine if it can limit the formation of fibrous scar tissue when administered by intradermal injection at the site of an excisional wound [90]. The phase II clinical trial is currently underway.

*MesomiR*

As in other cancer types, miRNA expression in malignant pleural mesothelioma (MPM) exhibits characteristic variations. The MesomiR 1 study is an ongoing phase I study testing the treatment of miR-15/16 (microRNAs implicated as tumour suppressors in MPM) packed in EDV TM nanocells (a 400 nm particle of bacterial origin able to carry a drug cargo) and targeted with EGFR antibodies (TargomiRs) in patients with MPM and refractory lung cancer (NSCLC) [91].

*MGN-1374*

miRagen Therapeutics is a company developing several miRNA-based drugs, among which ASO MGN-1374 targets the miR-15-family seed region. The 8-mer LNA oligonucleotide is in the preclinical stage for the control of postmyocardial infarction remodelling.

Of note, several other therapeutic miRNAs are under investigation.

## 5. Conclusions

Advances in understanding the role of miRNAs in pathophysiology, along with optimizing the efficacy and safety of anti-miRs/miRNA-mimic-based strategies, are contributing to the translation of miRNA research into clinical practice. RNA-based therapies have the remarkable potential to theoretically reach any relevant target, offering the opportunity to modulate specific miRNA levels. Currently, several RNA-based drugs have been approved for medical application, and the therapeutic potential of miRNA-based treatments is receiving increasing attention in relation to almost all human diseases, with the list of miRNA-based therapeutics in clinical trials expanding daily. Nevertheless, despite these exciting advancements, miRNA-based drugs have not yet passed phase III clinical trials and received approval from the US FDA for clinical use. In fact, several bottlenecks, such as best target selection, development of more effective modified ASOs, off-target binding, toxicity, immunogenicity, bioavailability, the efficiency of delivery systems to target organs, and vector encapsulation, still limit the possibility of translating miRNA-based approaches into therapeutic realities. Against this backdrop, numerous biotech and pharmaceutical companies are increasingly engaged in including miRNAs in their development pipeline to rapidly bring miRNA-based technology from the bench to the patient’s bedside.
